# Navigating the Debate on Managing Large (≥4 cm) Thyroid Nodules

**DOI:** 10.1155/2022/6246150

**Published:** 2022-04-16

**Authors:** Samantha N. Steinmetz-Wood, Amanda G. Kennedy, Bradley J. Tompkins, Matthew P. Gilbert

**Affiliations:** ^1^University of Vermont Medical Center, Burlington, VT, USA; ^2^Department of Medicine Quality Program, The Larner College of Medicine at The University of Vermont, Burlington, VT, USA

## Abstract

**Purpose:**

Discordant practice guidelines for managing large thyroid nodules may result in unnecessary surgeries and costs. Recent data suggest similar false-negative rates in fine needle aspiration (FNA) biopsies between small (<4 cm) and large (≥4 cm) nodules, indicating that monitoring rather than surgery may be appropriate for large biopsy-negative nodules. We investigated the management of thyroid nodules ≥4 cm to determine the proportion of surgeries not necessary for diagnostic purposes and examined for potential predictors.

**Methods:**

This was a retrospective cohort study of patients who received a FNA of nodule(s) ≥4 cm between 11/1/2014 and 10/31/2019 at the University of Vermont Medical Center. A surgery was considered unnecessary if the FNA result was benign in the absence of any of the following: compressive symptoms, family history of thyroid cancer in a first degree relative, history of neck irradiation, toxic nodule or toxic multinodular goiter, or substernal extension. Data were analyzed with Wilcoxon rank sum tests, chi square, or Fisher's exact tests.

**Results:**

177 patients had a ≥4 cm nodule during the timeframe and half (54.2%) had surgery. Patients who underwent surgery were significantly younger (51.5 years vs. 62 years; *P* < 0.001), more likely to report obstructive symptoms (34.4% vs. 12.1%; *P* < 0.001) and had a larger nodule size (5.0 cm vs. 4.7 cm; *P*=0.26). Forty-one patients with benign (Bethesda II) FNA results had surgery, all with negative surgical pathology. Thirteen percentage (23/177) of surgeries were potentially not necessary for diagnostic purposes.

**Conclusion:**

Approximately half of our patients with ≥4 cm nodules had surgery, with 13% having surgery not necessary for diagnostic purposes revealing opportunities for improving care and costs.

## 1. Background

Thyroid nodules are an increasingly common incidental finding in the general population, with the increased use of imaging technologies. They are especially common in older adults, females, and individuals with iodine deficiency or radiation exposure [1]. Studies estimate a prevalence of up to 6% by palpation, 19–68% with ultrasound, and 8–65% by autopsy [1–3]. Detecting and treating thyroid malignancy early in these nodules is ideal, but navigating algorithms for the management of thyroid nodules may pose complex clinical decisions for clinicians.

Fine needle aspiration (FNA) is a simple and effective procedure and is reported using the Bethesda System, established in 2007. Nodules categories (I-VI) range from nondiagnostic to malignant with their respective risks of malignancy [4]. Thyroid ultrasound (US) is also a simple and cost effective tool in both guiding FNA and in risk-stratifying nodules. The Thyroid Imaging, Reporting and Data System (TI-RADS) was introduced in 2015 to categorize nodules in a standardized fashion based on their US characteristics including composition, size, and echogenicity [5]. With the development of these standardized risk stratification systems and treatment algorithms, clinicians have become more skilled at navigating the management of thyroid nodules. This has helped standardize management but does not eliminate variability from the different skill levels of the clinicians performing biopsy, interpreting cytopathology, and interpreting US images. There is little debate about the management of small nodules <4 cm; however, there is disagreement about the management of ≥4 cm nodules. Some studies have shown false-negative results as high as 8 to 30% [6–12] and suggest surgery, or at the minimum intensive follow-up, as the most appropriate option [6, 8, 10, 11, 13, 14]. Many of these studies are limited by sampling error with some FNAs done by palpation, performed in different populations with different distributions of thyroid cancer, or were done prior to or not including current standardized radiology reporting systems (ACR TI-RADS) and cytology reporting systems (Bethesda). As a result, patients may undergo thyroidectomy/lobectomy for all nodules ≥4 cm, regardless of biopsy result.

However, increasingly more studies show much lower FNA false-negative rates, 0–5.2%, in large ≥4 cm nodules [15–25]. These studies suggest monitoring is more appropriate than surgery [15, 17–20, 22, 24–27]. In addition, a meta-analysis from 2018 found that there were not large enough differences in false-negative rates to support routine surgery [28].

The 2015 American Thyroid Association Management Guidelines for Adult Patients with Thyroid Nodules and Differentiated Thyroid Cancer states that if a nodule is benign on cytology, diagnostic studies or treatment are not required [29]. However, the guidelines express uncertainty about whether patients with thyroid nodules ≥4 cm and benign cytology carry a higher risk of malignancy and whether or not they should be managed differently from those with smaller nodules [29]. Discordant practice guidelines and data for managing large nodules may result in surgeries not necessary for diagnostic purposes, increased morbidity, and excess costs.

The purpose of this project was to describe the patients at the University of Vermont Medical Center (UVMMC) with ≥4 cm thyroid nodules and determine the proportion of patients who had surgery that was potentially not necessary for diagnostic purposes, predictors associated with unnecessary surgery, and the cost of unnecessary thyroid surgeries.

## 2. Methods

This was a retrospective cohort study of electronic health record data that included adult patients who received a FNA of nodule(s) ≥4 cm between 11/1/2014 and 10/31/2019 at UVMMC, a tertiary care institution located in Burlington, Vermont.

Thyroidectomies and partial thyroidectomies were performed by either the Otolaryngology or General Surgery services. If there was an incidental microcarcinoma outside the nodule in question, final surgical pathology was reported as benign. A surgery was considered unnecessary if the FNA result was benign in the absence of any of the following: compressive symptoms, family history of thyroid cancer in a first degree relative, history of neck irradiation, toxic nodule or toxic multinodular goiter, or substernal extension. Molecular testing results (using Afirma) were also collected for indeterminate (Bethesda III or IV) FNA samples when available.

Continuous variables were evaluated using the Wilcoxon rank-sum test, while categorical variables were tested using the chi-square or Fisher's exact test. All analyses were conducted using STATA 16.1 (Stata Corporation, College Station, TX). The financial data included the sum of all charges associated with the patient's account for their time in the hospital associated with their surgery, which included both the hospital billing (e.g., stay, supplies, and medications) and the professional billing from the surgeon for the surgery.

This study was approved by the University of Vermont Committees on Human Research in the Medical Sciences (clinical study registration number: STUDY00000783).

## 3. Results

A total of 177 patients had a ≥4 cm nodule and FNA during the time frame (Table 1). The majority of patients were women (68.4%) with a median age of 56.7 years. The median nodule size was 4.8 cm. Nearly a quarter of patients (23.2%) had documented obstructive symptoms.

Approximately half of patients (54.2%) with ≥4 cm nodules had surgery (Table 2). The 96 patients who underwent surgery were significantly younger (51.5 years vs. 62 years; *P* < 0.001), more likely to report obstructive symptoms (34.4% vs. 12.1%; *P*=0.001) and have a larger nodule size (5.0 cm vs. 4.7 cm; *P*=0.26) than patients who did not have surgery. The incidence of a clinically significant surgical pathology-confirmed thyroid cancer was approximately 23% (22/96). The majority of malignancies (63.6%) on final surgical pathology were papillary thyroid cancer (14/22) (Table 3). Forty-one patients with benign (Bethesda II) FNA results had surgery. All 41 patients were found to be negative at surgery, yielding a false-negative rate of 0.0% in this cohort. Twenty-three surgeries (24.0%) were considered unnecessary, and overall 13% (23/177) of patients with ≥4 cm nodules had potentially unnecessary surgery. There were no statistically significant differences in patient characteristics between surgeries considered appropriate versus unnecessary (Table 4). Charge data were available for 21 out of 23 patients who had a potentially unnecessary surgery. The median charge for these surgeries was $13,183 (IQR = $11,396–14,454).

A positive molecular testing result is considered a valid reason to get surgery, but due to availability, it was not done on all eligible patients with indeterminate biopsies. Fifty-two patients had an FNA result of atypia/follicular lesion of undetermined significance (Bethesda III) or follicular neoplasm/suspicious for follicular neoplasm (Bethesda IV). Fourteen of these patients (26.9%) had molecular testing completed.

## 4. Discussion

We found half of patients with large thyroid nodules ≥4 cm had surgery, especially patients who were younger, reported obstructive symptoms, and had larger nodule sizes. The prevalence of a clinically significant thyroid cancer confirmed by surgical pathology in the group of patients who had surgery was approximately 23% (22/96), which is comparable to the incidence reported in multiple other studies evaluating nodules ≥4 cm [8, 14, 18, 28]. Overall 13% of patients with ≥4 cm nodules had surgery that was potentially not necessary for diagnostic purposes. Incidentally the false-negative rate of patients who had a benign FNA and underwent surgery was 0.0%.

Early studies reported the false-negative rate as inappropriately high and supported surgical removal based on nodule size. These studies reported false-negative rates in the range of 8–20% for large thyroid nodules (≥3 cm or ≥4 cm) [6–12]. It is important to note that in these studies, not all preoperative FNAs were done under US guidance. Additionally, standardized stratification systems such as TI-RADS for ultrasound and the Bethesda system for cytopathology were not yet incorporated into the clinical decision making process [11]. Some more recently published studies such as this retrospective review of 648 patients who had undergone post FNA biopsy thyroidectomy in a single institution in Turkey between 2009 and 2014, also find a high false-negative rate of 24.2% for nodules >4 cm compared to 11.7% for smaller nodules. However, based on a smaller malignancy rate in large nodules (>4 cm) compared to smaller nodules (16.3% vs. 24.8%), as well as a higher specificity and accuracy in nodules larger than 4 cm, they support that nodule diameter alone should not be sufficient to outweight the increased morbidity and costs of thyroidectomy [30]. Another recent retrospective review of 103 patients with nodules ≥4 cm with preoperative FNA benign result who underwent thyroidectomy between 2010 and 2014 in a single institution in Korea found disproportionally high false-negative rate with 40 patients to have final malignant pathology and 63 patients to have benign pathology. It is unclear if some of these malignancies were clinically insignificant microcarcinoma. However, they did find that 42.5% of those with final malignant pathology had suspicious US findings [31].

Perspectives began to change with emerging research and US-guided FNAs. Porterfield et al. reported their results based on US-guided FNAs. Of the 145 patients with thyroid nodules ≥3 cm who underwent thyroidectomy, only one was false-negative. They also found no additional malignancies in 550 nodules with an average of 3 years of follow-up [16]. A prospective study published by Kuru et al. found the false-negative rate for nodules ≥4 cm to be 4.3% (4/98) compared to 1.3% (4/319) for nodules 4 cm. The authors considered the false-negative rate to be low and within an acceptable range, thus supporting avoiding thyroidectomy. All of the false-negatives identified were clinically insignificant microcarcinomas [17]. Rosario et al. reviewed 151 consecutive patients who had nodules ≥4 cm on US and who systematically had resection, regardless of cytopathology and find a false-negative rate of 3.6%, which they reported has changed management at their institution away from routine surgery for all nodules ≥4 cm [18]. A recent study by Kizilgul et al. did not find a significant difference in false-negative rates between nodules <4 cm and ≥4 cm (5.9% vs. 5.2%) [25].

Several long-term studies have confirmed the low false-negative rates of large nodules. A retrospective review of all FNAs performed during a 10-year period (2001–2011) at Walter Reed Army Medical Center reported that thyroid nodule size did not increase the risk of malignancy, nor did it increase significantly the risk of false-negative rate. Their false-negative rates were 7.0% overall and 7.1% in nodules ≥4 cm [27]. Bohacek et al. found similar results in a prospective review of 1000 FNAs with 451 nodules that underwent surgery at Cleveland Clinic between 2000 and 2010 and also find no significant increase in the rate of malignancy, nor in the false-negative rate with increasing size. They also noted a tendency towards a higher rate of malignancy in subcentimeter nodules [22]. This trend in subcentimeter nodules was also observed in other studies [23, 32], which puts into question the focus on large nodules. A retrospective cohort analysis by Kamran et al. 2013 of 4955 consecutive patients evaluated between 1995 and 2009 also found a low false-negative rate in nodules ≥4 cm of 1.3% and concluded that nodule size was not significantly associated with higher risk of false-negative aspirates compared to other size categories [19]. They also found that the risk and proportion of follicular carcinomas and Hurthle cell carcinomas increases with increased nodule size (diameter) and suggested that a change in the proportion of tumor type with increased size may in some instances be related to a change in malignancy risk and false-negative rates [19]. The increased proportion of follicular carcinomas in larger nodules was also demonstrated in other studies [32].

The primary focus of our study was the management of nodules at our academic medical center. Specifically, we sought to determine the proportion of surgeries for thyroid nodules ≥4 cm that were potentially not necessary for diagnostic purposes in light of the current evidence. We considered the recent body of evidence supporting the reliability and low false-negative rates of FNA in ≥4 cm nodules to be strong. Therefore, we considered surgery of biopsy benign nodules in the absence of a suspicious result on molecular testing, compressive symptoms, family history of thyroid cancer in a first degree relative, history of neck irradiation, toxic nodule or toxic multinodular goiter, or substernal extension to be unnecessary. Though a small sample size, our data demonstrated a reassuringly low false-negative rate of 0.0% and serves to show that there is room for improvement in avoiding potentially unnecessary surgeries. We support shared decision making with the patient about whether surgery is indicated, rather than basing the decision solely on nodule size.

There are several limitations to note. This was a single institution small study using retrospective data that included a largely rural and non-Hispanic white population. By study design, we did not include a comparison cohort of nodules <4 cm. During data collection, it was also evident that subjective reporting of compressive/obstructive symptoms can be difficult to quantify but cannot be discounted. In addition, it would be difficult to detect compressive symptoms reported after the clinical note which could have influenced the decision for surgery. By our data collection method, it would be difficult to capture patients' personal concerns of possible malignancy despite reassuring data on false-negative rates of FNA, and this was not included as a reason for appropriate surgery, nor did we include surgery for esthetic concerns. These reasons could have accounted for some of the surgeries that we marked as unnecessary. A positive molecular testing result would be a valid reason to get surgery, but due to availability, it was not done on all eligible patients with indeterminate biopsies. Ultimately, only surgery allows for final diagnosis and patients who want to make sure that there are no thyroid malignancies might prefer to undergo lobectomy for low suspicion nodules instead of the repeated US follow-up.

## 5. Conclusion

Approximately half of our patients with ≥4 cm nodules had surgery, especially patients who were younger, reported obstructive symptoms, and had larger nodule sizes. Of the patients who underwent surgery, the FNA false-negative rate was 0%. Overall 13% of our patients with ≥4 cm nodules had surgery for diagnosis that was potentially unnecessary revealing opportunities for improving care and costs.

## Figures and Tables

**Table 1:** Characteristics of patients who had a thyroid nodule ≥4.0 cm (*n* = 177).

Characteristic		
Age, median (IQR).	56.7	(44.4–66.7)
Female gender, ^#^ (%).	121	(68.4)
Family history of cancer, ^#^ (%)	11	(6.2)
History of radiation, ^#^ (%)	2	(1.1)
Obstructive symptoms, ^#^ (%)	41	(23.2)
Nodule size (cm), median (IQR).	4.8	(4.1–5.5)
Surgery, ^#^ (%)	96	(54.2)

**Table 2:** Characteristics of patients by surgery status.

Characteristic	No surgery (*n* = 81)	Surgery (*n* = 96)	*P*-value
Age, median (IQR)	62 (49.4–70.7)	51.5 (39.9–6 2.9)	<0.001
Female gender, ^#^ (%)	58 (71.6)	63 (65.6)	0.394
Family history of cancer, ^#^ (%)	3 (3.7)	8 (8.3)	0.231
History of radiation, ^#^ (%)	1 (1.2)	1 (1.0)	1.000
Obstructive symptoms^*∗*^, ^#^ (%)	8 (12.1)	33 (34.4)	0.001
Nodule size (cm), median (IQR)	4.7 (4.0–5.0)	5.0 (4.3)	0.026
Bethesda category^, ^#^ (%)			
Nondiagnostic or inadequate	4 (5.0)	3 (3.1)	<0.001
Benign	61 (76.3)	41 (42.7)	
Atypia/follicular lesion of undetermined significance	4 (5.0)	24 (25.0)	
Follicular neoplasm or suspicious for follicular neoplasm	8 (10.0)	16 (16.7)	
Suspicious for malignancy	0 (0.0)	5 (5.2)	
Malignant	3 (3.7)	7 (7.3)	

^*∗*^ *n* = 162 (15 patients reported “unknown” for the presence of obstructive symptoms). ^*n* = 176 (1 patient did not have a recorded Bethesda category).

**Table 3:** Type of malignancy on final surgical pathology by FNA result (*n* = 22).

	Malignancy type					
FNA result (bethesda category)	Follicular	Hurthle	Medullary	Papillary	Other	Total
Atypia/follicular lesion of undetermined significance (III)	2	0	0	3	0	5
Follicular neoplasm or suspicious for follicular neoplasm (IV)	3	1	0	2	0	6
Suspicious for malignancy (V)	0	0	0	4	0	4
Malignant (VI)	0	0	1	5	1^*∗*^	7
*Total*	5	1	1	14	1	22

^*∗*^ *n* = 1 patient with final surgical pathology showing renal cell carcinoma.

**Table 4:** Characteristics of patients by whether a surgery was considered appropriate for diagnosis.

Characteristic	Appropriate Surgery (*n* = 73)	Surgery not necessary for diagnosis (*n* = 23)	*P*-value
Age, median (IQR)	48.6 (39.2–62.7)	54.6 (50.4–65.8)	0.089
BMI, median (IQR)	29.5 (25.4–34.7)	29.4 (27.4–32.7)	0.604
Female gender, ^#^ (%)	49 (67.1)	14 (60.9)	0.582
Family history of cancer, ^#^ (%)	8 (11.0)	0 (0.0)	—
History of radiation, ^#^ (%)	1 (1.4)	0 (0.0)	—
Obstructive symptoms^*∗*^, ^#^ (%)	33 (45.2)	0 (0.0)	—
Nodule size (cm), median (IQR)	4.9 (4.2–5.6)	5.0 (4.5–6.0)	0.053
Positive surgery result, # (IQR)	22 (30.1)	0 (0.0)	—

## Data Availability

Restrictions apply to the availability of some or all data generated or analyzed during this study to preserve patient confidentiality.
